# Editorial: The Origin of Plant Chemodiversity – Conceptual and Empirical Insights

**DOI:** 10.3389/fpls.2020.00890

**Published:** 2020-06-30

**Authors:** Kazuki Saito

**Affiliations:** ^1^RIKEN Center for Sustainable Resource Science, Yokohama, Japan; ^2^Plant Molecular Science Center, Chiba University, Chiba, Japan

**Keywords:** phytochemicals, plant metabolism, metabolomics, biosynthesis, specialized metabolite, evolution, chemodiversity, plant chemicals

Whenever one looks plant -made chemicals, everyone is fascinated with their huge chemodiversity, being estimated at over one million metabolites (Afendi et al., 2012), and then impressed how people lives rely on those plant chemicals as food, drugs, flavors, cosmetics, and industrial raw materials. Although it is generally accepted that such vast plant chemodiversity is the consequence of the evolutional history of plants as sessile organisms, the queries regarding the origin of plant chemodiversity are not fully addressed.

By taking the advantage of recently advanced theory and technology of genomics and associated fields (Rai et al., 2017), such as comparative genomics, metabolomics, bioinformatics and molecular evolution, one may address the questions mentioned above quite directly. Besides answering such fundamental questions, the knowledge gained will contribute to the development of sustainable society, which is formulated as 2030 Agenda for Sustainable Development and its 17 Sustainable Development Goals (United Nations, 2018).

Only outstanding experts in this field were invited to contribute their articles to this Research Topic. The collected papers widely cover the topic from conceptual discernment to empirical insights. Finally, in total, 23 articles were published and divided into several categories: 9 in Review, 2 in Mini Review, 2 in Perspective, 1 in Hypothesis and Theory, 8 in Original Research, and 1 in Correction.

Metabolomics has now maturely held an indispensable position to decipher the chemodiversity of plants. However, peak annotation of acquired data by untargeted analysis is still challenging. Rutz et al. report an improved method for annotation of natural products with taxonomic information. Spatio-temporal metabolite and elemental profiling were applied to investigate salt-stress response in barley (Gupta et al.). The impacts of natural climate and geography on the metabolome of tobacco is reported by Ma et al.. The paper by Šamec et al. deals with the metabolome of the Psilotales, which exhibit unique anatomical character in the fern lineage.

Several articles deal with an evolutionary consideration in the expansion of plant chemodiversity. Two Perspective articles discuss on evolution aspects: The report by Kusano et al. provides an evolutional perspective regarding two transferases often involved in plant secondary (recently often referred to as “specialized”) metabolism; another perspective paper by Shirai and Hanada discusses the effects of copy number variations on functional divergence in the cross-species and inside-species diversity. Shoji proposes the evolutional model based on recruitment of genes by focusing on jasmonate-responsive transcription factors. The Mini-Review article by Maeda provides insight on the evolutional diversification of plant primary metabolism concerning the robustness of plant metabolism and further diversification in specialized metabolism.

Two papers deal with the novel concept and approach to extend our understanding of plant metabolism. The Hypothesis and Theory article by Schwachtje et al. discusses the imprinting and priming plant metabolism against upcoming environmental challenges as a novel concept for adaptation and diversification of plant metabolism responding to stresses. To obtain further insights on mechanisms of plant natural products biosynthesis, computational chemistry may play a central role—Sato et al. present an overview of cutting-edge aspects of application of computational chemistry on the biosynthetic mechanisms of plant specialized metabolites.

The rest of the 13 articles focuses on relatively the pathway-specific metabolism or metabolites with some generalization across the biosynthetic pathways. Four papers deal with flavonoids and phenylpropanoids. Yonekura-Sakakibara et al. discuss the origin of flavonoid biosynthetic enzymes with functional genomics implication in their comprehensive Review Article. Another Review Article (Davies et al.) provides the current information about the flavonoid pathway in the bryophytes (liverworts, hornworts, and mosses). The role of the flavonoid metabolons formed by specific protein-protein interactions of the biosynthetic enzymes is discussed in the Review Article (Nakayama et al.). The Original Research Article by Cheevarungnapakul et al. reports the genes for the biosynthesis of caffeoylquinic acids in sunflower.

Terpenoids form the largest sub-family of specialized metabolites with their immense possibility in the expansion of structural diversity. The Review Article by Karunanithi and Zerbe provides up-to-date knowledge on the functional diversity and molecular evolution of the plant terpene synthases widely across the plant kingdom. The Original Article contributed by Muchlinski et al. reports biosynthesis and emission of mixtures of monoterpenes and sesquiterpenes triggered by the generalist herbivore in Switchgrass. Three papers focus on triterpenoid saponins. Lei et al. studied the large-scale profiling of saponins in 201 ecotypes of *Medicago truncatula*. The Original Article by Fanani et al. reports the detailed investigation on the molecular basis for C-30 oxidation of triterpenoids leading to the production of high-value saponins such as glycyrrhizin in Legume licorice. Cárdenas et al. in their Mini-Review Article discuss convergent and divergent evolution in triterpenoid biosynthesis, and the mechanisms increasing structural diversity within and across plant species.

Three contributions dealing with nitrogen- and sulfur-containing specialized metabolites are included. Morris and Facchini(a); Morris and Facchini(b) provide an overview of the functional diversification of methyltransferases in the biosynthesis of benzylisoquinoline alkaloids. The Review by Chhajed et al. discusses the cellular and subcellular organization of the glucosinolates -myrosinase system, its chemodiversity and functions in different cell types, emphasizing single-cell-type studies. Sugiyama and Hirai provide an up-to-date information on the diversity and the role of atypical myrosinases beyond the classical model of glucosinolates -myrosinase system.

Overall, this Research Topic becomes an excellent anthology to exhibit state-of-the-art on the theme of Topic by contributions from world-experts of this field. The Research Topic can play a role as a sort of flagship topic of the Specialty Section of Plant Metabolism and Chemodiversity. The curiosity-driven fundamental research on the origin and evolution of diversification of plant chemicals is primarily essential. Besides, the basic knowledge obtained could contribute to solving the current global problems such as climate crisis and pandemic diseases. I hope this Research Topic could be a landmark of future research in this field.

## Author Contributions

KS contributes entirely to the paper.

## Conflict of Interest

The author declares that the research was conducted in the absence of any commercial or financial relationships that could be construed as a potential conflict of interest.
